# Green Manure Amendment Can Reduce Nitrogen Fertilizer Application Rates for Oilseed Rape in Maize–Oilseed Rape Rotation

**DOI:** 10.3390/plants10122640

**Published:** 2021-12-01

**Authors:** Chiming Gu, Wei Huang, Yue Li, Yinshui Li, Changbin Yu, Jing Dai, Wenshi Hu, Xiaoyong Li, Margot Brooks, Lihua Xie, Xing Liao, Lu Qin

**Affiliations:** 1 Key Laboratory of Biology and Genetic Improvement of Oil Crops, Oil Crops Research Institute of the Chinese Academy of Agricultural Sciences, Ministry of Agriculture of China, Wuhan 430062, China; guchiming@foxmail.com (C.G.); liyue1231122@126.com (Y.L.); lysh@webmail.hzau.edu.cn (Y.L.); cbyu123@163.com (C.Y.); daijing@caas.cn (J.D.); huws@webmail.hzau.edu.cn (W.H.); dashuai_17@163.com (X.L.); xielh@oilcrops.cn (L.X.); 2 Huanggang Academy of Agricultural Sciences, Huanggang 438000, China; hw_hgaas@163.com; 3 Department of Biochemistry and Microbiology, Rhodes University, Grahamstown 6140, South Africa; brooksmargs@yahoo.co.uk

**Keywords:** green manure, nitrogen application, chemical fertilizer reduction, oilseed rape, maize–oilseed rape rotation

## Abstract

Excessive use of chemical fertilizers has led to a reduction in the quality of arable land and environmental pollution. Using green manure to replace chemical fertilizers is one of the most effective solutions. To study the effect of green manure on the requirement for nitrogen fertilizer in oilseed rape, a field experiment with maize–oilseed rape rotation was conducted. Green manure was intercropped between rows of maize and returned after the maize harvest, with no green manure intercropped as control. Different nitrogen fertilizer treatments (0, 65%, 75% and 100% N rates, respectively) were applied during the oilseed rape season. The results showed that with a 35% reduction in nitrogen application rate, the rapeseed grain yield was significantly higher with the maize intercropping with green manure returned to the field than with the maize monocropping treatment at the same nitrogen level. Under conditions of intercropping and return of green manure, compared with the full standard rate of nitrogen fertilizer treatment, a reduction in nitrogen application of 25–30% in the rape season had no significant effect on rape yield. The agronomic efficiency of nitrogen fertilizer on oilseed rape increased significantly, by 47.61–121%, with green manure incorporation. In addition, green manure incorporation significantly increased the soil organic matter content and the soil-available nitrogen content when chemical nitrogen fertilization was abandoned. Benefit analysis showed that a 25–35% reduction in chemical nitrogen fertilizer applied to oilseed rape crops could be achieved by intercropping green manure in the maize season before the sowing of rapeseed in the experimental area. In the long-term, this measure would increase nitrogen utility, reduce production costs, and have concomitant environmental benefits of improving the quality of cultivated land.

## 1. Introduction

Consequential to the continuous increase in the global population, the demand for agricultural products is increasing rapidly. In order to increase yields, the amount of chemical fertilizer used in agricultural production is escalating, while the application of organic fertilizer remains insufficient. The cultivability of arable land deteriorates under long-term chemical fertilizer application, and environmental pollution is prominent [1,2,3]. The necessity for increasing the input of organic fertilizer is especially urgent worldwide [4,5,6,7].

**Figure 1:**
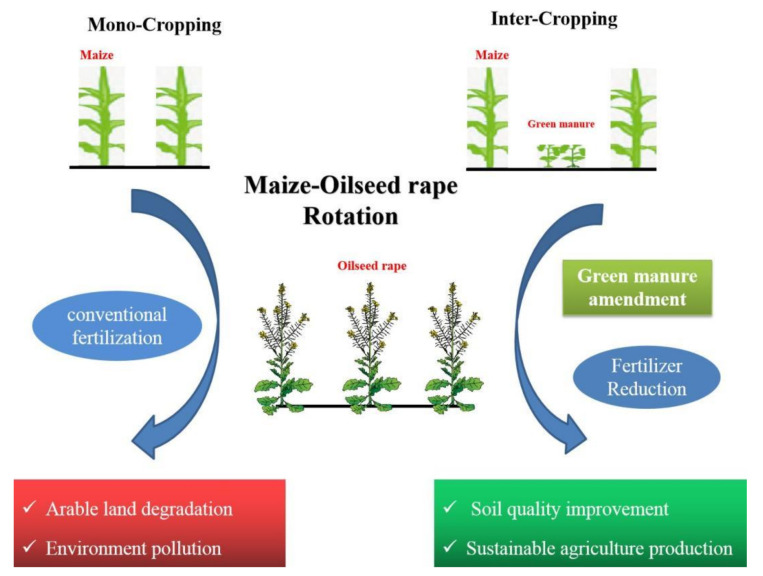
Green manure amendment in maize–oilseed rape rotation.

Intercropping green manure is a productive planting system that can make full use of sunlight and heat resources, soil moisture, and nutrients. It is a practical measure to produce and utilize green manure and contribute to sustainable agricultural production [8,9,10]. It is estimated that 15–20% of food production in the world has involved intercropping [11,12], and it has been widely used in China for centuries [13,14]. Intercropping with legumes has a crucial advantage as legumes can fix nitrogen from the air to supply extra nitrogen to the soil for crops, and as high-quality green manure, it is beneficial for soil quality when legume biomass is returned to the field [15,16] (Figure 1).

Oilseed rape is the source of the world’s third most consumed edible oil after soybean and palm and is also the primary source of biodiesel [17]. In 2018, the planting area of rapeseed in China was 6.55 million hectares, and the average annual output of rapeseed oil was 6.6 million tons, making it the largest source of edible oil in China [18]. Thus, oilseed rape has a pivotal role in ensuring food security, and increasing the production of rapeseed oil is of strategic significance for ensuring the global supply of edible oil [19,20].

Maize–oilseed rape rotation is widely practiced as a crop rotation system worldwide [8,21]. In this rotation system, intercropping green manure between rows of maize can offer multiple benefits. It rationalizes the planting configuration, facilitates full utilization of resources such as light, heat, water, and nutrients, maximizes the output potential of the land while also providing high-quality green manure for the oilseed rape [22]. In addition, the effect of biological nitrogen fixation by the legumes and the reduction of soil surface evaporation by covering the ground [23]; both present effective ways to reduce the application of chemical fertilizers and increase the seed yield of oilseed rape [24].

However, there are few reports of research on the potential reduction in the requirement for chemical fertilizer that may be achieved by intercropping with legumes in the preceding crop season and supplying green manure for the later season’s oilseed rape crop in the maize–oilseed rape rotation systems. We hypothesized that:

(i) legume Labadou (*Lablab purpureus* (L.) Sweet) facilitates reduction in the need for fertilizer-N in intercropping systems of maize and oilseed rape;
(ii) growing Labadou in the summer-autumn growing season increases biomass production and fertilizer-N efficiency of winter oilseed rape (*Brassica napus* L.);
(iii) introducing an intercropping method to maize and oilseed rape rotation systems in dryland decreases N application rates.

To test these hypotheses, a maize–oilseed rape rotation field trial was conducted to study the effects of different treatments on the yield of corn and rapeseed and basic soil properties. Maize intercropping with leguminous green manure (IC) and maize monocropping (MC) planting configurations were set up for the maize season, and different chemical nitrogen doses were set based on the standard local rapeseed nitrogen application rate (N, 169 kg ha^−1^).

## 2. Results

### 2.1. Analysis of the Meteorological Data of the Experimental Site

**Figure 2:**
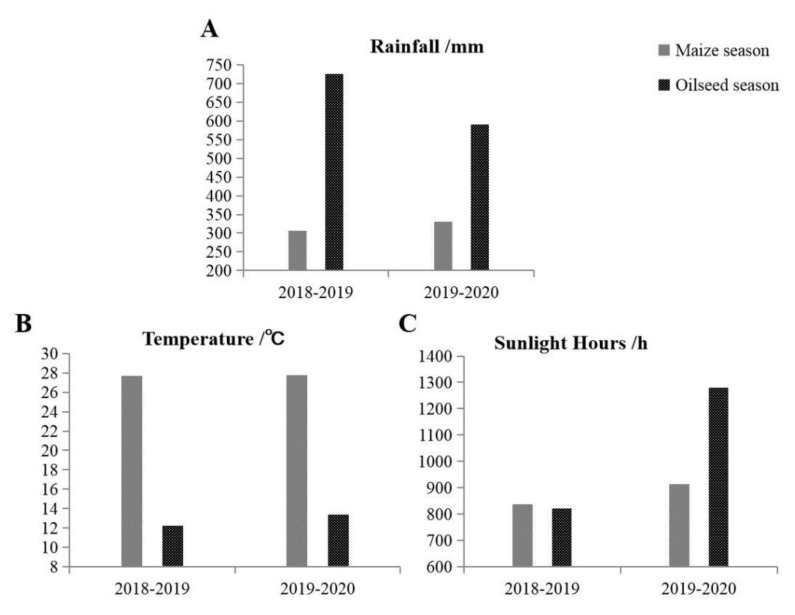
Basic meteorological data of the experimental site during experimental period. (**A**): Rainfall; (**B**): Temperature; (**C**): Sunlight Hours.

The rainfall, temperature, and duration of sunshine at the experimental site during the experimental periods are shown in Figure 2. Comparing the two maize seasons (from June to September of each year), the total rainfall, average temperature, and total sunshine hours in 2019 were higher than in 2018. Rainfall, temperature, and total sunshine duration increased by 7.78%, 0.37%, and 9.34%, respectively. For the two oilseed rape seasons (from October to May of the following year), the average temperature and total sunshine duration of the second oilseed rape season (2019–2020) was significantly higher, increasing by 9.40% and 55.64%, respectively, compared to the first season (2018–2019), and the total rainfall was significantly lower, reduced by 18.82%. As the rainfall in the 2019 maize season was 23.9 mm higher than in 2018. Still, the average temperature difference between the two seasons was only 0.1 °C, the soil moisture for the second rapeseed season was better than for the first season. While 63.86% of the total decrease in total rainfall occurred in the second oilseed rape season, the rapeseed was planted with suitable moisture content and did not suffer water damage after emergence.

### 2.2. Effects of Different Treatments on Crop Yield

**Figure 3:**
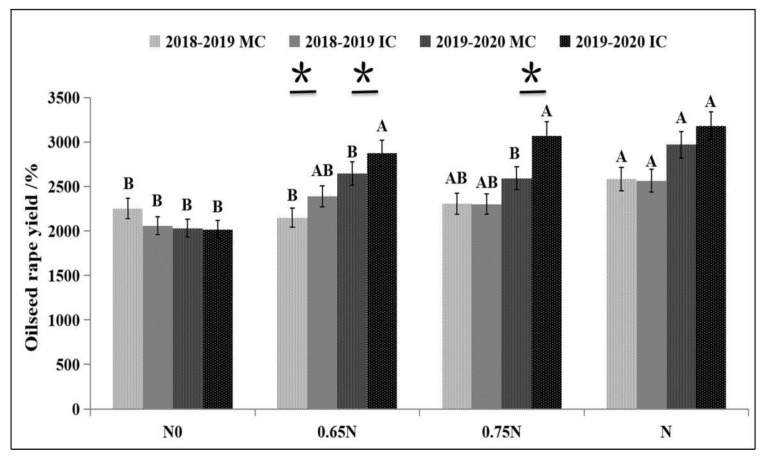
Effects of different treatments on the grain yield of oilseed rape (kg ha^−1^). Note: * Represents the significant difference between MC and IC at the same nitrogen dose in the same year; capital letters indicate the difference between treatments with different nitrogen doses in the same year and the same mode.

During the experimental period, compared with MC, the maize yield of IC showed no significant difference. Intercropping with green manure, or not, during the maize season had no significant effect on the yield of both maize and rapeseed. Green manure yield in IC reached averages of 22.03 t ha^−1^ (Table 1). The effect of green manure combined with different nitrogen application rates on oilseed rape yield is shown in Figure 3. During the experimental period, the rapeseed yield in MC increased significantly with increasing nitrogen application. Compared with N0, N application significantly increased the yield of rape in MC, however, there was no significant difference between different N application rates in IC.

In the first experimental season (2018–2019), compared with MC + N, the rapeseed yield was significantly reduced by 20.31% in MC + 0.65N, while no significant differences were found under IC. In the second season (2019–2020), compared with MC + N, the rapeseed yield was significantly reduced by 20.12% and 14.51% in MC + 0.65N and MC + 0.75N, respectively (Figure 3). However, there was still no statistically significant decrease found in IC + 0.65N and IC + 0.75N. The rapeseed grain yield with 0.65N was significantly higher in IC than in MC + 0.65N by 11.32% and 8.77%, respectively, in the first and second seasons. In IC + 0.75N, yield increased significantly by 18.44% in the second season. The yield components of maize showed no significant differences between MC and IC. No trends were found in oilseed rape component yield among different treatments (Table 2).

Results of the analysis of the nitrogen fertilizer agronomic efficiency of rapeseed under different treatments are shown in Table 3. The nitrogen fertilizer agronomic efficiency of oilseed rape with IC was significantly higher than with MC in the two consecutive seasonsby 121% and 47.61% in the first and second seasons, respectively.

### 2.3. Effects of Different Treatments on Soil Properties

**Figure 4:**
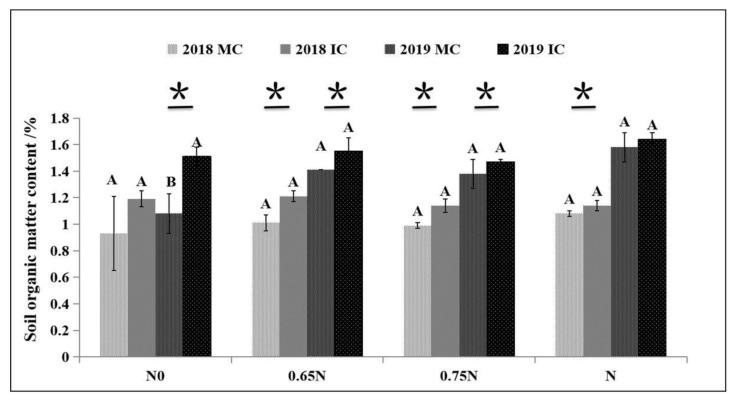
Effects of different treatments on soil organic matter content (%). Note: * Represents the significant difference between MC and IC at the same nitrogen dose in the same year; different capital letters indicate the difference between treatments with different nitrogen doses in the same year and the same mode; bars below letters represent standard deviation.

The effect of green manure under different nitrogen application rates on soil organic matter content is shown in Figure 4. Compared with MC, IC could increase soil organic matter by 27.96% and 39.81% in N0, 19.80% and 9.93% in 0.65N, 15.15% and 6.52% in 0.75N, and 5.56% and 3.80% in N in the first and second seasons, respectively (Figure 4). These changes reached significant levels in the first and second seasons in 0.65N and 0.75N. The differences in soil pH between different treatments are shown in Table 4. Under both planting modes, the pH value of N0 was the highest. However, in this study, the nitrogen application rate and the rotation method had no significant effect on the soil pH (Table 4).

The differences in the content of soil-available nitrogen between different treatments are shown in Table 5. With an increase in the nitrogen application rate, the soil-available nitrogen content in the same planting mode showed a significant increase. When nitrogen was not applied, the soil-available nitrogen in IC was significantly higher than in MC, for the full duration of the experiment. When the nitrogen dose was reduced by 35%, the soil-available nitrogen content in IC was higher than that in MC both in 2018 and 2019, but this was not significant. The 0.75N and N treatments showed no apparent trends.

The differences in soil-available phosphorus content between different treatments are shown in Table 6. The data showed that both cropping mode and nitrogen fertilizer dose had no significant effect on the soil-available phosphorus content during the entire experimental period.

The difference in soil-available potassium content between different treatments is shown in Table 7. With the increase in nitrogen application rate, the soil-available potassium in MC showed a trend of significantly decreasing. A similar decreasing trend could be seen in IC, but this trend did not reach a significant level. When the full standard nitrogen dose was applied, soil-available potassium was significantly higher in IC than in MC. Under the same dose reduction in nitrogen application, the cropping mode had no significant effect on the soil-available potassium content.

## 3. Discussion

### 3.1. Effects of Intercropping Combined with Nitrogen Application on Crop Growth

Existing studies have shown that rapeseed yield may vary according to the different effects of previous crop types [25,26,27]. In our maize-rapeseed rotation study, intercropping green manure had no significant effect on maize yield. However, it significantly reduced the nitrogen fertilizer required in subsequent oilseed rape crops. Under the condition of 35% nitrogen reduction, the average yield of oilseed rape after IC was significantly higher than after maize monocropping, by 10.05%. Because of root nodule nitrogen fixation and the nitrogen transfer capability of leguminous plants, and the concomitant suppression of the soil weed seed bank to decrease competition with crops for nutrients [28], legume intercropping with gramineous crops can promote the absorption and utilization of nitrogen in gramineous crops and significantly increase yield [22]. Compared with monocropping, intercropping legume green manure increased the yield of proso millet by 13.9–50.1% [29] and maize by 35% [22]. As reported, approximately two-thirds of 11 rice cultivars [30] and black oats [31] showed significantly increased yields with intercropping, compared with monocropping.

However, many researchers have found different results. In our study, even with a 25–35% reduction in nitrogen fertilizer, the rapeseed yield was not significantly different from the yield with the standard nitrogen dose when under the maize-intercropping mode, indicating that green manure in the previous maize intercropping could substitute 25–35% of nitrogen fertilizer for oilseed rape production in the experimental site.

The amount of N transferred from a legume to associated crops is a subject of considerable controversy. It varies depending on conditions that impact legume N-fixation, such as legume species, symbiotic performance, and agronomic factors, such as weather conditions [32]. In our research, total rainfall, average temperature, total sunshine hours, and especially rainfall distribution, influenced oilseed rape seedling growth and yield (Figure 2). In this experiment, the legume green manure in the maize–oilseed rape rotation also significantly impacted the oilseed rape nitrogen utilization. Compared with monocropping, the agronomic efficiency of rapeseed nitrogen fertilizer utilization under intercropping could be significantly increased by 121% and 47.61% in the first and second seasons, respectively.

The application rate of nitrogen fertilizer has a considerable influence on the yield and quality of rapeseed. Frugal application of nitrogen fertilizer has a critical role in saving production costs and improving the yield and quality of oilseed rape [33]. At present, the existing research on green manure pertains mainly to grain crops or others with higher economic value [22,24,31,34]. Studies have rarely associated the application of green manure with the reduction of nitrogen fertilizer and nitrogen utilization efficiency of oilseed rape. Prior studies found that compared with the sole application of chemical fertilizers, combining with green manure significantly increased the nitrogen accumulation and the nitrogen fertilizer agronomic efficiency of spring maize [16] and rice [35].

### 3.2. Effects of Intercropping Combined with Nitrogen Application on Soil Properties

In this experiment, after two years of continuous planting and return of green manure, the soil organic matter significantly increased by 3.80% to 39.81%. The effect of planting and returning legume green manure on soil properties is closely related to the soil property background value [35]. It has been found that organic matter accumulates faster in soil with a lower background organic matter value after green manure amendment, while in the short term (1–2 years), returning green manure has no apparent impact on soil with a higher organic matter background value [35,36]. The soil in this study had a relatively low organic matter background value of about 1.46%. Thus, with a lower background value, soil organic matter significantly increased. Generally, the long-term application of green manure results in a significant increase in soil organic matter, but in the short term, the effect of turning green manure into the field varies due to differences in soil, climate, and farming systems [37,38]. In addition, whether green manure amendment has any significant impact on soil organic matter depends upon the duration of application, the varieties, and the dose of green manure, among other factors [39,40]. In general, because the soil’s available nutrients change rapidly in the soil, they will be affected by many factors, including farming methods, fertilizer types, fertilizer application methods, crop growth, and rainfall [37,38,40]. Thus, the analysis of changes in soil-available nutrients must consider multiple factors.

Studies have shown that green manure amendment can increase the content of available nitrogen [34], available phosphorus [37,41], and available potassium [1] in soil. In our study, when nitrogen fertilizer application was abandoned, the soil-available nitrogen of IC increased significantly for the entire duration of the experiment. However, there were no obvious trends for soil pH, soil-available phosphorus, and potassium content. The input of green manure or organic fertilizer can increase the activity of soil microorganisms [35,42], which in turn activates the soil’s nitrogen, phosphorus, and potassium nutrients and micronutrient elements [41,43,44], leading to increased yield [45]. In some studies, even a reduction in the amount of chemical fertilizer applied had no significant effect on the soil nutrient content of the farmland after green manure amendment [36,46]. Interestingly, in our study the input of green fertilizer had no effect on phosphorus and potassium content, and the precise reason needs to be discovered in the subsequent work.

### 3.3. Benefit Analysis

We calculated the benefit under different treatments based on local maize and oilseed rape prices and labor costs. The results of the benefit analysis showed that although the intercropping would increase seed and labor costs, the overall benefit of intercropping would still be slightly higher than monocropping due to a reduction in the nitrogen fertilizer application dose by 25–35% (Table 8). More importantly, the intercropping would also increase the soil organic matter content and improve the soil quality. There would be concomitant environmental benefits in the long term, due to the improved quality of cultivated land.

## 4. Materials and Methods

### 4.1. Experimental Site

The experimental site is located in Meijiadun Village, Ducheng Town, Huangzhou District, Huanggang City, Hubei Province, China. It is 114°88′ east longitude, 30°43′ north latitude, and 26.7 m altitude. It has a typical humid subtropical monsoon climate, with abundant water and heat resources. The rainfall is mainly concentrated from April to August. The annual average rainfall is (1287 ± 305) mm, the annual average temperature is (17.7 ± 0.5) °C, the annual average sunshine duration is 1900 h, and the frost-free period is 260 d. The soil texture is sandy loam and had the following chemical characteristics: SOM was 1.46%, pH (H_2_O: soil = 5:1) was 7.53, alkali-hydrolyzable nitrogen was 61.43 mg kg^−1^, available phosphorus was 5.07 mg kg^−1^, and available potassium was 101.87 mg kg^−1^.

### 4.2. Experimental Design

**Figure 5:**
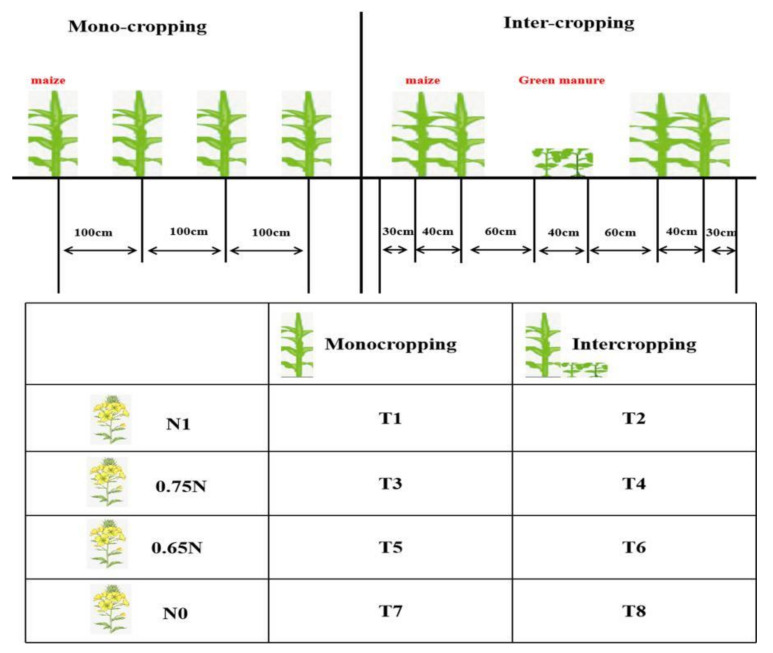
Schematic diagram of maize planting mode and treatment design. Note: T1, T3, T5, T7 represents maize monocropping combined with 100%, 75%, 65%, 0 nitrogen doses in oilseed rape, respectively; T2, T4, T6, T8 represents maize intercropping combined with 100%, 75%, 65%, 0 nitrogen doses in oilseed rape.

The experimental design was based on the split zone test. The primary strategy was to compare the effect of green manure intercropping with monocropping, while the secondary strategy was to measure this interaction with varying fertilizer doses (only for oilseed rape). For the green manure intercropping treatment (IC), wide and narrow row spacings were adopted in the maize season, and Labadou (*Lablab purpureus* (L.) Sweet), leguminous green manure, was interplanted between the wide rows of maize (*Zea mays* L.), the variety of maize is zhengdan69, was grown for grain. The width between maize rows was 160 cm and between plants was 15 cm for the wide rows and 40 cm × 15 cm for the narrow rows. The row spacing for the maize monocropping treatment (MC) was 100 cm × 15 cm (Figure 5). The sowing rate of maize was 37.5 kg ha^−1^, and the density was approximately 55,000 plants ha^−1^. Maize was planted within half a month after the previous rapeseed crop was harvested. Oilseed rape (*Brassica napus* L.) was direct sown at a rate of 5.25 kg ha^−1^. The density was approximately 35,000 plants ha^−1^, and the variety of oilseed rape is yangguang 2009. The green manure was sown at the same time as the maize, with two rows of Labadou (40 cm × 8 cm) sown between the wide maize rows. The nutrient content of 100 kg Labadou was tested to be 2.33 kg N, 0.30 kg P_2_O_5_, and 2.53 kg K_2_O.

The chemical fertilizer used in the experiment was urea (46% N); superphosphate (12% P_2_O_5_) and potassium chloride (60% K_2_O). The same amount of fertilizer was applied in all plots in the maize season (N:P_2_O_5_:K_2_O was 195:90:135 kg ha^−1^). In the oilseed rape season, the application rate of P_2_O_5_, K_2_O, and Boron fertilizer in all plots was 60, 75 and 7.5 kg ha^−1^, respectively. The application rate of nitrogen fertilizer was divided into four levels: full standard nitrogen application (N, 169 kg ha^−1^), 25% nitrogen reduction (0.75 N, 126.75 kg ha^−1^), 35% nitrogen reduction (0.65 N, 109.85 kg ha^−1^) and no nitrogen fertilizer (N0, 0 kg ha^−1^) (Figure 5). Three replicates entailed a total of 24 plots. Labadou was returned with the maize straw into the 20 cm deep soil layer after the maize was harvested as green manure. The full standard quantities of phosphorus and potassium fertilizers were used as base fertilizer, while 60% nitrogen fertilizer was used as base fertilizer and 40% applied as topdressing in the budding stage. Field trials with summer maize-winter oilseed rape rotations were carried out from 2018 to 2020.

### 4.3. Sampling and Analysis

Each year, mixed soil samples were collected from the soil surface layer (0–20 cm) at five points in each plot, using a soil borer, after the oilseed rape was harvested. After removing all animal and plant residues and pebbles, soil samples were air-dried and put through a 100 or 200 mesh sieve for further analysis. Soil properties were determined as follows: total soil organic matter (SOM) using the K_2_Cr_2_O_7_ oxidation method; available N using the Alkaline diffusion method; available phosphorus (AP) concentration using the Olsen method after extraction with 0.03 mol L^−1^ NH_4_F 0.025 mol L^−1^ HCL; available K using flame photometry after extraction with 1 mol L^−1^ NH_4_OAC, and pH (1:10, soil to water rate) using a pH meter [47]. The agronomic efficiency of treatments was calculated using the following equation:

$${\mathrm{AE}}_{i}=\left(Y_{i}-Y_{\mathrm{CK}}\right)/N_{i}$$

AE_i_ represents the Agronomic efficiency of treatment i, Y_i_ represents the yield of treatment i, Y_CK_ represents the yield of treatment CK, N_i_ represents the nitrogen fertilizer application dose of treatment i.

Benefit analysis was calculated as follows: maize and oilseed rape production income, seed and fertilizer cost were calculated according to local agriculture market prices. Labor cost was calculated according to local labor market standards. Total income was the sum of maize and oilseed rape production income; the benefit is total income minus total cost.

### 4.4. Statistical Analyses

Experimental data were analyzed using the SPSS 16.0 software package and Microsoft Office Excel 2010. Analysis of variance (ANOVA) was carried out to determine the differences between the measured parameters for different treatments. The least significant difference (LSD) at *p* = 0.05 was used to elucidate any significant differences.

## 5. Conclusions

This study provided a better understanding of green manure utilization in the intercropping planting system to support more effective nitrogen fertilization in the maize–oilseed rape rotation system. Strategies that reduce fertilizer application while maintaining yield are vital for food production methodologies that guarantee agricultural sustainability, particularly in the drought rotation area. Intercropping legume green manure, Labadou, during the maize season and returning it to the field before rapeseed sowing showed obvious substitution effects for rapeseed nitrogen fertilizer. The results of two consecutive years of field trials showed that 25–35% of nitrogen fertilizers could be replaced by 19–24 t ha^−1^ of legume green manure, a precondition ensuring the seed yield of rapeseed in the experimental site. Intercropping and returning can significantly increase the soil organic matter content of the experimental site and intercropping Labadou with maize is a promising sustainable and low-cost method to improve soil fertility.

## Figures and Tables

**Table 1:** Yield of maize, oilseed rape (kg ha^−1^) and Labadou (t ha^−1^).

Treatment	2018–2019		2019–2020	
	MC	IC	MC	IC
Maize	5483 ± 49.81a	5425 ± 131.92a	5484 ± 311a	5185 ± 102a
Oilseed rape	2322 ± 186a	2302 ± 310a	2558 ± 390a	2785 ± 527a
Labadou	-	19.99 ± 1.76	-	24.07 ± 2.69

Note: Different lowercase letters represent the differences between MC and IC.

**Table 2:** Yield components of maize and oilseed rape under different treatments.

Treatment			Maize			Oilseed Rape		
			Ears per ha	Kernels per Ear	Mass per 1000 Kernels (g)	Pods per Plant	Seeds per Pod	Mass per 1000 Seeds (g)
2018–2019	MC	N0	3.55a	572a	270.05a	301aA	15.87bA	3.61aA
		0.65N				314aA	15.88bA	3.83aA
		0.75N				304aA	16.68aA	3.71aA
		N				313aA	16.05aA	3.8aA
	IC	N0	3.46a	565a	277.33a	295aA	16.59aAB	3.54aB
		0.65N				250bAB	16.73aA	3.82aA
		0.75N				265bA	16.99aA	3.76aA
		N				242bB	16.4aB	3.98aA
2019–2020	MC	N0	3.32a	534a	309.195a	118aB	21.33aA	3.77aA
		0.65N				158aA	20.47bB	3.80aA
		0.75N				148bAB	21.67aA	3.76aA
		N				175aA	20.67aAB	3.82aA
	IC	N0	3.17a	521a	313.94a	143aB	20.87aA	3.75aB
		0.65N				195aA	21.80aA	4.01aA
		0.75N				204aA	20.40bB	3.77aA
		N				190aA	21.00aA	3.90aA

Note: Different lowercase letters represent the significant difference between MC and IC at the same nitrogen dose in the same year; different capital letters indicate the difference between treatments with different nitrogen doses in the same year and the same planting mode.

**Table 3:** Nitrogen fertilizer agronomic efficiency of oilseed rape under different treatments (kg kg^−1^).

Treatment	2018		2019		Average
	MC	IC	MC	IC	
N0	-	-	-	-	-
0.65N	-	2.26	4.20	5.88	4.11
0.75N	0.32	1.43	3.33	6.24	2.83
N	1.47	2.24	4.17	5.15	3.26
average	0.90b	1.98a	3.90b	5.76a	

Note: Different lowercase letters represent the significant difference between MC and IC in the same year.

**Table 4:** Effects of different treatments on soil pH.

Treatment	2018		2019	
	MC	IC	MC	IC
N0	7.53 ± 0.45aA	7.67 ± 0.35aA	7.54 ± 0.27aA	7.77 ± 0.20aA
0.65N	7.29 ± 0.26aA	7.44 ± 0.31aA	7.43 ± 0.26aAB	7.37 ± 0.24aB
0.75N	7.25 ± 0.06aA	7.57 ± 0.31aA	7.22 ± 0.06bB	7.56 ± 0.28aAB
N	7.50 ± 0.29aA	7.48 ± 0.26aA	7.50 ± 0.29aA	7.51 ± 0.25aAB

Note: Different lowercase letters represent the significant difference between MC and IC at the same nitrogen dose in the same year; different capital letters indicate the difference between treatments with different nitrogen doses in the same year and the same planting mode.

**Table 5:** Effects of different treatments on soil-available nitrogen content (mg kg^−1^).

Treatment	2018		2019	
	MC	IC	MC	IC
N0	61.92 ± 2.37bB	78.99 ± 6.63aA	63.65 ± 2.43bB	73.86 ± 8.90aAB
0.65N	67.83 ± 3.69aB	71.18 ± 2.05aB	71.91 ± 3.79aAB	73.17 ± 2.10aB
0.75N	65.94 ± 1.42aB	76.54 ± 10.84aAB	78.98 ± 19.30aAB	78.44 ± 7.88aAB
N	71.96 ± 1.42bA	81.00 ± 8.36aA	83.49 ± 16.52aA	80.97 ± 9.87aA

Note: Different lowercase letters represent the significant difference between MC and IC at the same nitrogen dose in the same year; different capital letters indicate the difference between treatments with different nitrogen doses in the same year and the same planting mode.

**Table 6:** Effects of different treatments on soil-available phosphorus content (mg kg^−1^).

Treatment	2018		2019	
	MC	IC	MC	IC
N0	8.18 ± 0.88aA	7.85 ± 4.19aA	7.82 ± 0.88aA	7.49 ± 2.48aA
0.65N	8.50 ± 2.64aA	9.48 ± 0.60aA	7.02 ± 2.65aA	9.13 ± 1.56aA
0.75N	8.71 ± 2.39aA	9.78 ± 0.77aA	7.38 ± 2.40aA	9.43 ± 1.57aA
N	8.32 ± 1.29aA	9.54 ± 4.89aA	8.71 ± 1.29aA	9.19 ± 0.63aA

Note: Different lowercase letters represent the significant difference between MC and IC at the same nitrogen dose in the same year; different capital letters indicate the difference between treatments with different nitrogen doses in the same year and the same planting mode.

**Table 7:** Effects of different treatments on content of soil-available potassium (mg kg^−1^).

Treatment	2018		2019	
	MC	IC	MC	IC
N0	194 ± 51.42aA	210 ± 28.14aA	195 ± 50.94aA	211 ± 36.87aA
0.65N	178 ± 35.30aA	211 ± 32.18a	179 ± 34.97aAB	211 ± 33.22aA
0.75N	167 ± 30.37bAB	206 ± 12.54aA	185 ± 30.07aA	207 ± 14.61aA
N	154 ± 13.7bB	194 ± 28.57aA	163 ± 13.57bB	195 ± 30.54aA

Note: Different lowercase letters represent the significant difference between MC and IC at the same nitrogen dose in the same year; different capital letters indicate the difference between treatments with different nitrogen doses in the same year and the same planting mode.

**Table 8:** Analysis of benefits (CNY ha^−1^).

Treatment		Production		Total Income	Cost				Benefits
		Maize	Oilseed Rape		Seed	Fertilizer	Labor	Other	
IC	0.65N	10,967	7187	18134	850	3450	1500	500	11,834
	0.75N		7347	18294	850	3524	1500	500	11,920
	N		8327	19274	850	3707	1500	500	12,717
MC	0.65N	10,610	7899	18509	1000	3450	1650	500	11,909
	0.75N		8055	18665	1000	3524	1650	500	11,991
	N		8610	19220	1000	3707	1650	500	12,363

## Data Availability

We would like to exclude this statement as the study does not report any online available data.
